# Impact of Self-Contained Underwater Breathing Apparatus Diving on French Military Divers’ Lung Function: A Retrospective Cohort

**DOI:** 10.1016/j.chpulm.2025.100158

**Published:** 2025-03-24

**Authors:** Justin Ulm, Jean-Eric Blatteau, Luc Aigle, Roman Glogowski, Olivier Castagna, Arnaud Druelle

**Affiliations:** a Emergency Department, Percy Military Teaching Hospital, Clamart, France; b Department of Diving and Hyperbaric Medicine, Sainte-Anne Military Teaching Hospital, Toulon, France; c Ecole Militaire de Santé de Lyon Bron, Direction des Etudes, Bron, France; d Department of Anesthesiology and Intensive Care, Bethesda Hospital, Duisburg, Germany; e Underwater research team (ERRSO), Military Biomedical Research Institute (IRBA), Toulon, France; f LAHMESS (UPR 6312), Université de Nice, Nice, France

**Keywords:** diving, long-term impact, lung function, military

## Abstract

**Background:**

Although acute risks concerning underwater divers are well documented, the literature regarding the long-term impacts on pulmonary function remains inconclusive. Although oxygen toxicity at high partial pressures is established for patients in intensive care, the impact of diving seems limited.

**Research Question:**

What are the long-term effects of self-contained underwater breathing apparatus diving on pulmonary function in military divers?

**Study Design and Methods:**

This longitudinal study used routinely collected data from Sainte-Anne Military Teaching Hospital, Toulon, France, over a 20-year follow-up period. We expressed spirometric measures as a percentage of predicted values to account for age, height, and ethnicity, and analyzed them using mixed-effects models. The impact of diving is assessed for different gas and diving apparatus. Other included variables were atopy, tobacco use, and BMI.

**Results:**

A total of 331 divers were included (2,685 spirometric measurements), with an average follow-up of 23.9 years and 2,491 dives. Only male divers met inclusion criteria. Baseline FEV_1_ matched predicted values (100.00%; SD, 11.98). Every 1,000 dives, FEV_1_ increased by 3.21% (95% CI, 2.73-3.68; *P* < .001), regardless of gas or apparatus. FVC (3.02%; 95% CI, 2.52-3.53; *P* < .001) and forced expiratory flow when 75% of forced expiratory vital capacity has been exhaled (10.12%; 95% CI, 8.29-11.95; *P* < .001) increased, whereas FEV_1_/FVC remained stable. Each BMI point increase was associated with a 0.51% rise in FVC (*P* = .010) and 0.38% rise in FEV_1_ (*P* = .032), whereas each pack-year was associated with a 1.12% decline in forced expiratory flow when 75% of forced expiratory vital capacity has been exhaled (*P* = .005).

**Interpretation:**

Our results show that self-contained underwater breathing apparatus diving is associated with increased pulmonary flows and volumes in this population of military divers. Prospective studies could explore the role of unmeasured confounding factors and could significantly contribute to health policies for both military and civilian divers.

**Take-Home Points**

**Study Question:** What are the long-term effects of self-contained underwater breathing apparatus diving on pulmonary function in military divers?

**Results:** In this study, diving history was shown to be associated with higher pulmonary flows and volumes in this population of military divers.

**Interpretation:** Our findings emphasize the importance of further investigating the long-term effects of underwater diving on pulmonary function among military personnel.

To breathe underwater, divers use self-contained underwater breathing apparatus (SCUBA) equipment. In most cases, air is used, which contains about 20% oxygen. According to the Dalton law, as the depth of immersion increases, the partial pressure of oxygen increases. Therefore, even when the diver breathes air, their pulmonary system is exposed to hyperoxia. During certain technical dives, whether civilian or military, divers use oxygen-enriched mixture containing nitrogen and up to 60% oxygen (NITROX), or even pure oxygen (100%).^1^ In these cases, exposure to hyperoxia is even more significant. The toxicity of prolonged exposure to high concentrations or partial pressures of oxygen has been demonstrated in patients hospitalized in ICUs, where it can lead to potentially fatal pulmonary fibrosis.^2^ The consequences include impaired gas exchange at the alveolar level, measured by the carbon monoxide transfer factor, and a decrease of FEV_1_/FVC.^2^^,^^3^

Furthermore, military and professional divers use specific breathing equipment that increases the mechanical strain on the lungs.^4^^,^^5^ For example, the use of dorsal rebreathers can cause pulmonary edema due to differences in hydrostatic pressure between the lungs of the divers and the apparatus. However, the impact of prolonged and repeated exposure to a hyperbaric environment, as imposed by underwater diving, remains to be clarified.^3^^,^^6^ A literature review from 2014 aiming to highlight the impact of underwater diving on ventilatory function found 15 articles with a longitudinal design^7^: sample sizes were small, follow-up durations were short, and results were contradictory. Overall, it suggests a small deterioration in ventilatory function with a decrease in FEV_1_,^8^, ^9^, ^10^, ^11^ peripheral flows (forced expiratory flow when 50% of forced expiratory vital capacity has been exhaled [FEF_50__%_], forced expiratory flow when 75% of forced expiratory vital capacity has been exhaled [FEF_75%_]),^8^^,^^9^^,^^12^ FVC,^9^, ^10^, ^11^ and carbon monoxide transfer factor.^12^, ^13^, ^14^, ^15^ Among these studies, only 3 found a significant decrease in FEV_1_ after age-standardization.^8^^,^^10^^,^^12^ Thus, this literature review does not allow us to conclude whether the variations in spirometric parameters are caused by diving exposure or ageing,^7^ and whether these minimal variations may have clinical consequences. Finally, these studies did not consider the gas and type of diving apparatus used. More recently, 2 wider retrospective studies with 232 and 1,260 divers concluded that diving had no clinically significant impact on lung function.^16^^,^^17^

Considering the data demonstrating the toxicity of high partial pressure oxygen on lung function and given the varying conclusions of previous studies on the long-term impacts among professional divers, we aimed to determine whether the professional practice of underwater diving could permanently affect the ventilatory function of French military divers. More precisely, we wonder whether practicing professional military diving for > 20 years permanently alters the ventilatory function of military divers. If so, are there identifiable risk factors individually or related to diving practice and the gas mixtures used?

## Study Design and Methods

### Study Design and Setting

We conducted a retrospective monocentric study using routinely collected hospital data on a cohort of military SCUBA divers who were followed-up at Sainte-Anne Military Teaching Hospital, (Toulon, France), where they attend fitness-to-dive assessments every 4 years. This fitness-to-dive assessments include spirometric measurements.

### Participants

All French military divers are followed up at Sainte-Anne Military Teaching Hospital with spirometry data currently available in the database. Depending on their specialty, these divers may use mainly an open circuit diving apparatus with air, an open circuit diving apparatus with NITROX, a semiclosed circuit diving apparatus with NITROX, or a closed-circuit diving apparatus with pure oxygen.

We included all divers with a follow-up beginning before January 1, 2002, to assess the long-term impact of diving on lung function with a manageable sample. Divers were excluded if spirometric measures at baseline (free of exposure to military diving) were missing; no spirometric measurement was conducted at 20 years of follow-up or after; or data on birthdate, height at admission, or history of SCUBA diving in civilian structures before admission were missing.

Because the study was conducted in a military setting, the project was presented to and approved (No. 2022HJ29/EPSMPP) by the French Armed Forces Health Service. A detailed data management plan was developed with the French Armed Forces Health Service data controller representative, and the research was conducted according to the principles of the World Medical Association Declaration of Helsinki. Patient consent was not required, but patients were informed of their right to withdraw.

### Data Set, Variables, and Measurements

The available data include spirometric measurements (FEV_1_, FVC, FEF_75%_, forced midexpiratory flow), diving exposure such as the type of apparatus and mixture used (air, NITROX, oxygen, open-circuit, semiclosed, or closed-circuit-rebreather), and the number of dives for each of these apparatuses and mixtures. Also collected were the following: age, sex, height, weight, diver’s professional specialty, military branch, and comorbidities (eg, atopic history, diving accident history, smoking pack-years). Spirometry tests were conducted by nursing staff. The measurement tools varied throughout the follow-up period, with changes in the spirometer model and brand, software, and calibration protocol. We calculated predicted values for all spirometric values included, using the Global Lung Intiative 2012 equations.^18^^,^^19^ These equations, developed by the Global Lung Initiative, were published in 2012 to provide reference equations accross spiromery devices and to develop a standardized approach to interpreting lung function. New variables, expressed as % predicted values, allowed us to account for the natural evolution of lung function over time by comparing our measured data with a control population.

### Study Size

Previous longitudinal studies mainly have included small samples of < 30 patients, except for 1 study, which included 1,260 patients with a mean follow-up time of 5 years.^20^ These studies have conflicting results and do not allow us to safely estimate the effect of diving on spirometric parameters. Thus, we were not able to calculate a minimal sample size for our own study.

### Analysis

All analyses were performed in R software (version 4.2.2; R Core Team) and RStudio (2022.07.2+576; RStudio Team). The primary outcome was the change in FEV_1_ (as a percentage of predicted value) per 1,000 dives, calculated separately for each dive type (compressed air open circuit, NITROX open circuit, NITROX semiclosed circuit, and oxygen closed circuit). The model adjusts these coefficients for clinical characteristics (eg, atopic history, tobacco use, BMI), resulting in 4 distinct coefficients representing the change in FEV_1_ for each type of dive. Secondary outcomes include changes in FVC, FEF_75%_, and FEV_1_/FVC (as a percentage of predicted value) per 1,000 dives. Parametric *t* tests, paired and unpaired, were used after controlling for normality to assess differences between quantitative variables. For qualitative variables, the χ^2^ test was used. Before any quantitative analysis, a test for equality of variances (Fisher test) was conducted. If this test indicated significantly different variances, the Welch test was used to compare means; otherwise, the Student *t* test was applied. Results are reported as mean ± SD for normally distributed data or as median and interquartile range for nonnormally distributed data, with a 95% CI. The chosen significance threshold was *P* < .05.

A multiple linear model with mixed effects was used to evaluate the impact of diving on pulmonary function. Fixed effects included duration of diving exposure in years, presence of atopic history, tobacco use status expressed in pack-years, and diving characteristics. Random effects were considered at the diver level to account for individual differences among divers. This model allows for considering unmeasured parameters and estimating the ventilatory function evolution for each group and an average slope based on all divers. Each diver could dive with > 1 apparatus. In the model, the number of dives for each apparatus was accounted (ie, diver may have dived 100 times with a closed circuit and 500 times with an open circuit at the time of a fitness-to-dive assessment). Both values were included in the model. Missing data, representing < 10% of the sample, were disregarded. Otherwise, multiple imputation would have been considered.

## Results

### Participants

**Figure 1:**
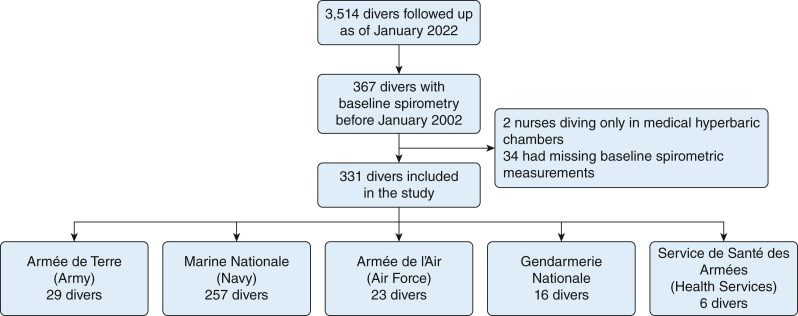
Flowchart of inclusion of eligible patients.

Among approximately 3,500 divers who underwent follow-up, 367 were followed up for > 20 years and were screened for inclusion. A total of 36 divers were excluded for reasons presented in Figure 1. There were 331 divers finally included in the statistical analysis, representing 2,685 spirometric evaluations. All were male. Five different army branches are represented, each corresponding to different operational diving constraints.

### Descriptive Data

Sociodemographic characteristics are presented in Table 1. The divers had an average age of 24.1 ± 2.8 years at the beginning of their career. Previous experience in recreational diving before their professional career was reported by 119 divers (37.7%), with 37 (11.7%) having experience of > 100 dives. For 15 divers, no information on dives before their professional career was available. The average duration of follow-up was 23.9 ± 5.5 years, with a maximum of 42.9 years. At the end of the follow-up, 70 divers (20.7%) were reservists. The total number of dives averaged 2,469 ± 1,201, with a maximum of 7,060. Of the divers, 170 (52.6%) primarily dived with compressed air, 45 (13.9%) used closed-circuit rebreathers, 47 (14.6%) used open-circuit with NITROX, and 60 (18.2%) regularly used semiclosed-circuit rebreathers.

**Table 1:** Sociodemographic Characteristics of Participants at Baseline and End of Follow-Up

Characteristic	Value	Range
Age at baseline, y	24.1 [2.8]	17-35
Rank category		
Officer	34 (10.7)	
Warrant officer	285 (89.3)	
No. of dives preceding military training	68 [186]	0-1,500
0	197 (62.3)	
1-100	82 (25.9)	
> 100	37 (11.7)	
No. of dives at end of follow-up	2,469 [1,201]	450-7,060
< 1,001	14 (4.5)	
1,001-2,000	123 (39.5)	
2,001-3,000	92 (29.6)	
> 3,000	82 (26.4)	
Main apparatus and gas mixture used during follow-up		
Air open circuit	170 (52.6)	
NITROX open circuit	47 (14.6)	
NITROX semiclosed-circuit rebreather	61 (18.9)	
Oxygen closed-circuit rebreather	45 (13.9)	
No. of dives with air open circuit	1,273 [1,505]	200-6,000
No. of dives with NITROX open circuit	237 [505]	0-3,200
No. of dives with NITROX semiclosed-circuit rebreather	564 [1,112]	0-6,000
No. of dives with oxygen closed-circuit rebreather	420 [979]	0-6,050

Values are presented as No. (%) or mean [SD]. NITROX = oxygen-enriched mixture containing nitrogen and up to 60% oxygen.

The average weight of the divers at baseline was 73.3 ± 7.3 kg (95% CI, 72.5-74.1) and 80.1 ± 9.4 kg (95% CI, 79.1-81.1) at the end of the follow-up period; the difference was significant (*P* < .001). BMI was 23.2 ± 1.7 kg/m^2^ (95% CI, 23.0-23.4) at baseline and 25.4 ± 2.3 kg/m^2^ (95% CI, 25.2-25.7) at the end of follow-up (*P* < .001). Tobacco use, atopy, respiratory comorbidities, and spirometric values are summarized in Table 2. A total of 24 diving accidents were reported, including decompression sickness (n = 5), pulmonary edema (n = 5), barotrauma (n = 4), and biochemical accidents (hypoxia, hyperoxia, hypercapnia, narcosis) (n = 10).

**Table 2:** Clinical Characteristics of Participants at Baseline and End of Follow-Up

Characteristic	Baseline	End of Follow-Up	*P* Value
Tobacco use			**< .001**
Does not smoke	261 (79.1)	235 (71.2)	
Previously used tobacco	61 (18.5)	43 (13.0)	
Active tobacco use	8 (2.4)	52 (15.8)	
Smoking consumption (pack-years)	2.2 [1.3]	10.2 [7.2]	**< .001**
BMI, kg/m^2^	23.2 [1.7]	25.4 [2.3]	**< .001**
< 25	259 (85.2)	138 (45.1)	
≥ 25 kg/m^2^	45 (14.8)	168 (54.9)	
Atopic/allergic history	53 (16.6)	53 (16.6)	> .99
Known respiratory comorbidities	5 (1.6)	17 (5.3)	**.009**
Any other known medical condition	5 (1.6)	40 (12.5)	**< .001**
Currently on daily medication	1 (0.3)	13 (4.1)	**.001**
FVC, L	5.4 [0.7]	5.4 [0.9]	.703
FVC, % predicted value	98.6 [11.5]	108.4 [15.2]	**< .001**
FEV_1_, L	4.6 [0.6]	4.3 [0.6]	**< .001**
FEV_1_, % predicted value	100.0 [11.9]	109.2 [14.9]	**< .001**
FEV_1_/FVC, %	85.3 [6.6]	80.0 [6.1]	**< .001**
FEV_1_/FVC, % predicted value	101.7 [7.7]	101.1 [7.7]	.358
FEF_75%_, L	2.7 [0.9]	1.9 [0.6]	**< .001**
FEF_75%_, % predicted value	122.1 [39.7]	148.7 [50.5]	**< .001**
FEF25-75%, L	5.2 [1.1]	4.2 [1.0]	**< .001**
FEF25-75%, % predicted value	107.3 [22.4]	115.7 [28.5]	**< .001**

Values are presented as No. (%), mean [SD], or as otherwise indicated. Values that are presented in bold font have been determined to be statistically significant. FEF25-75% = forced midexpiratory flow; FEF_75%_ = forced expiratory flow when 75% of forced expiratory vital capacity has been exhaled.

### Main Results

#### Models’ Conception

**Figure 2:**
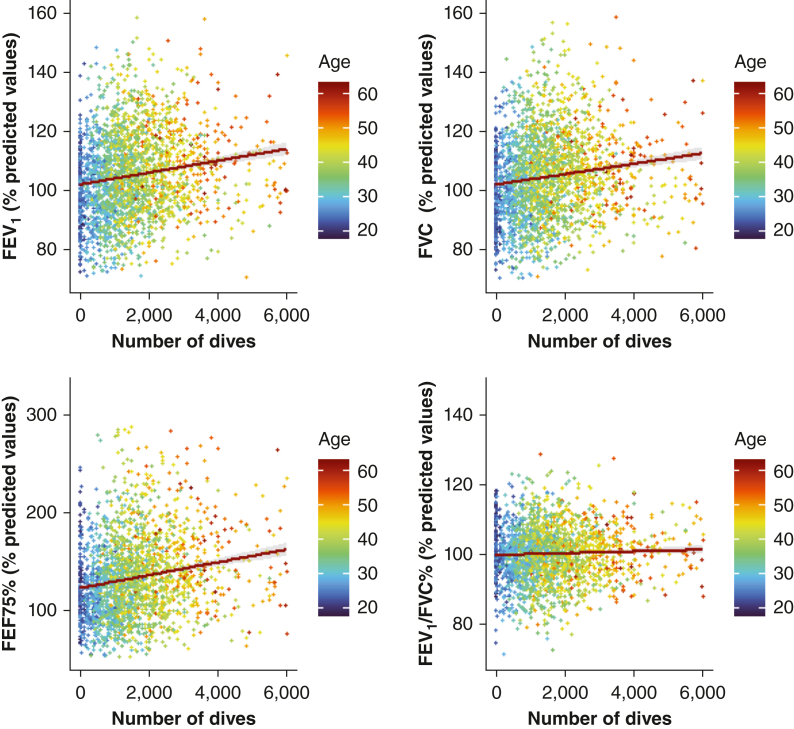
Evolution of spirometric values according to the number of dives and age at the time of data collection. FEF75% = forced expiratory flow when 75% of forced expiratory vital capacity has been exhaled.

The regression models’ intraclass correlation coefficients are all between 0.40 and 0.70, confirming a cluster effect and justifying the use of mixed-effects models. Diagnostic tests for each mixed-effects regression model showed that the models satisfy the necessary conditions for validation. Table 3, Table 4, Table 5, Table 6 present detailed results of each model, and Figure 2 summarizes association between the number of dives and FVC, FEF_75%_, and FEV_1_ as a percentage of predicted values.

**Table 3:** Multivariate Analysis of FEV_1_ (% Predicted Values) (n = 309)

Characteristic	Univariate			Multivariate			Complete Model		
	ß	95% CI	*P* Value	ß	95% CI	*P* Value	ß	95% CI	*P* Value
Total No. of dives^a^	2.21	1.70 to 2.71	**< .001**	3.21	2.73 to 3.68	**< .001**	NA	NA	NA
No. of air open circuit^a^	2.62	2.06 to 3.19	**< .001**	NA	NA	NA	3.42	2.80 to 4.04	**< .001**
No. of NITROX open circuit^a^	1.15	−0.51 to 2.81	.175	NA	NA	NA	3.83	2.14 to 5.53	**< .001**
No. of NITROX semiclosed circuit^a^	−0.27	−0.98 to 0.43	.443	NA	NA	NA	2.98	2.10 to 3.86	**< .001**
No. of oxygen closed circuit^a^	0.52	−0.31 to 1.36	.220	NA	NA	NA	2.42	1.38 to 3.47	**< .001**
Rank category^b^			.795			.197			.165
Officer	−0.21	−1.82 to 1.39		1.41	−0.73 to 3.55		1.52	−0.63 to 3.67	
BMI	0.93	0.66 to 1.20	**< .001**	0.36	0.02 to 0.70	**.039**	0.38	0.03 to 0.72	**.032**
Pack-year	−0.22	−0.39 to −0.05	**.010**	−0.07	−0.30 to 0.16	.546	−0.07	−0.30 to 0.16	.526
Atopic history	2.67	1.22 to 4.13	**< .001**	1.78	−1.58 to 5.15	.297	1.65	−1.72 to 5.01	.336

No. of observations = 2,386; sigma = 8.85; intraclass correlation coefficient = 0.60; log-likelihood = −8.965; Akaike information criterion = 17,951; Bayesian information criterion = 18,015; restricted maximum likelihood criterion = 17,929; conditional *R*^2^ = 0.627. Values that are presented in bold font have been determined to be statistically significant. NA = not applicable; NITROX = oxygen-enriched mixture containing nitrogen and up to 60% oxygen.

a Estimates for the number of dives (total and per gas/apparatus) represent the evolution of FEV_1_ per 1,000 dives.

b Baseline category = warrant officer.

**Table 4:** Multivariate Analysis of FEF_75%_ (% Predicted Values) (n = 308)

Characteristic	Univariate			Multivariate			Complete Model		
	ß	95% CI	*P* Value	ß	95% CI	*P* Value	ß	95% CI	*P* Value
Total No. of dives^a^	7.39	5.78 to 9.00	**< .001**	10.12	8.29 to 11.95	**< .001**	NA	NA	NA
No. of air open circuit^a^	6.83	5.04 to 8.62	**< .001**	NA	NA	NA	11.49	9.21 to 13.78	**< .001**
No. of NITROX open circuit^a^	4.80	−0.40 to 10.00	.070	NA	NA	NA	14.41	7.97 to 20.86	**< .001**
No. of NITROX semiclosed circuit^a^	1.47	−0.76 to 3.69	.195	NA	NA	NA	6.83	3.55 to 10.11	**< .001**
No. of oxygen closed circuit^a^	1.46	−1.18 to 4.11	.227	NA	NA	NA	7.93	3.95 to 11.91	**< .001**
Rank category^b^			.644			.746			.515
Officer	−1.22	−6.38 to 3.94		1.16	−5.88 to 8.20		2.35	−4.73 to 9.44	
BMI	1.48	0.59 to 2.38	**.001**	0.64	−0.55 to 1.84	.290	0.56	−0.64 to 1.77	.357
Pack-year	−0.99	−1.52 to −0.46	**< .001**	−1.13	−1.91 to −0.35	**.004**	−1.12	−1.90 to −0.34	**.005**
Atopic history	4.56	−0.15 to 9.28	.058	0.51	−8.47 to 9.50	.911	−0.16	−9.17 to 8.84	.972

No. of observations = 2162; sigma = 32.6; intraclass correlation coefficient = 0.41; log-likelihood = −8.965; Akaike information xriterion = 21,731; Bayesian information criterion = 21,777; restricted maximum likelihood criterion = 21,715; conditional *R*^2^ = 0.457. Values that are presented in bold font have been determined to be statistically significant. FEF_75%_ = forced expiratory flow when 75% of forced expiratory vital capacity has been exhaled; NA = not applicable; NITROX = oxygen-enriched mixture containing nitrogen and up to 60% oxygen. Values that are presented in bold font have been determined to be statistically significant.

a Estimates for the number of dives (total and per gas/apparatus) represent the evolution of FEV_1_ per 1,000 dives.

b Baseline category = warrant officer.

**Table 5:** Multivariate Analysis of FVC (% Predicted Values) (n = 309)

Characteristic	Univariate			Multivariate			Complete Model		
	ß	95% CI	*P* Value	ß	95% CI	*P* Value	ß	95% CI	*P* Value
Total No. of dives^a^	1.98	1.48 to 2.48	**< .001**	3.02	2.52 to 3.53	**< .001**	NA	NA	NA
No. of air open circuit^a^	2.07	1.50 to 2.63	**< .001**	NA	NA	NA	3.65	2.94 to 4.36	**< .001**
No. of NITROX open circuit^a^	3.15	1.50 to 4.80	**< .001**	NA	NA	NA	3.57	1.55 to 5.58	**< .001**
No. of NITROX semiclosed circuit^a^	−0.36	−1.06 to 0.34	.310	NA	NA	NA	3.85	2.80 to 4.90	**< .001**
No. of oxygen closed circuit^a^	0.70	−0.13 to 1.53	.098	NA	NA	NA	2.99	1.70 to 4.28	**< .001**
Rank category^b^			.792			.548			.677
Officer	0.22	−1.38 to 1.81		0.68	−1.54 to 2.90		0.48	−1.80 to 2.77	.22
BMI	0.97	0.70 to 1.24	**< .001**	0.48	0.12 to 0.84	**.009**	0.51	0.12 to 0.89	**.010**
Pack-year	−0.07	−0.24 to 0.09	.391	−0.08	−0.31 to 0.16	.527	−0.09	−0.35 to 0.16	.479
Atopic history	2.42	0.97 to 3.87	**.001**	1.38	−1.79 to 4.54	.392	1.50	−1.79 to 4.79	.369

No. of observations = 2,386; sigma = 9.61; intraclass correlation coefficient = 0.54; log-likelihood = −9.120; Akaike information criterion = 18,256; Bayesian information criterion = 18,302; restricted maximum likelihood criterion = 18,240; conditional *R*^2^ = 0.577. Values that are presented in bold font have been determined to be statistically significant. NA = not applicable; NITROX = oxygen-enriched mixture containing nitrogen and up to 60% oxygen.

a Estimates for the number of dives (total and per gas/apparatus) represent the evolution of FEV_1_ per 1,000 dives.

b Baseline category = warrant officer.

**Table 6:** Multivariate Analysis of FEV_1_/FVC (% Predicted Values) (n = 309)

Characteristic	Univariate			Multivariate			Complete Model		
	ß	95% CI	*P* Value	ß	95% CI	*P* Value	ß	95% CI	*P* Value
Total No. of dives^a^	0.19	−0.07 to 0.46	.158	0.11	−0.17 to 0.40	.434	NA	NA	NA
No. of air open circuit^a^	0.46	0.16 to 0.76	**.003**	NA	NA	NA	0.16	−0.23 to 0.56	.417
No. of NITROX open circuit^a^	−1.99	−2.85 to −1.12	**< .001**	NA	NA	NA	0.23	−0.88 to 1.34	.686
No. of NITROX semiclosed circuit^a^	0.14	−0.22 to 0.51	.440	NA	NA	NA	−0.32	−0.89 to 0.25	.266
No. of oxygen closed circuit^a^	−0.16	−0.60 to 0.27	0.461	NA	NA	NA	0.05	−0.64 to 0.75	.883
Rank category^b^			.393			.520			.279
Officer	−0.37	−1.20 to 0.47		0.39	−0.83 to 1.60		0.68	−0.55 to 1.91	
BMI	−0.06	−0.20 to 0.09	.443	−0.08	−0.27 to 0.12	.444	−0.12	−0.33 to 0.09	.271
Pack-year	−0.14	−0.23 to −0.05	**.002**	−0.04	−0.16 to 0.09	.591	−0.05	−0.19 to 0.08	.433
Atopic history	0.09	−0.68 to 0.85	.825	0.24	−1.35 to 1.83	.763	0.19	−1.44 to 1.83	.815

No. of observations = 2,386; sigma = 5.49; intraclass correlation coefficient = 0.45; log-likelihood = −7.750; Akaike information criterion = 15,517; Bayesian information criterion = 15,563; restricted maximum likelihood criterion = 17,929; conditional *R*^2^ = 0.454. NA = not applicable; NITROX = oxygen-enriched mixture containing nitrogen and up to 60% oxygen. Values that are presented in bold font have been determined to be statistically significant.

a Estimates for the number of dives (total and per gas/apparatus) represent the evolution of FEV_1_ per 1,000 dives.

b Baseline category = warrant officer.

##### FEV_1_

FEV_1_ significantly increased with the number of dives, with an average of 3.21% of predicted values for each 1,000 dives (95% CI, 2.73-3.68; *P* < .001). There was no significant difference in using 1 gas mixture over another or using different apparatuses. FEV_1_ increased with BMI (0.38%; 95% CI, 0.03-0.72; *P* = .032).

##### Forced Expiratory Flow When 75% of Forced Expiratory Vital Capacity has Been Exhaled

FEF_75%_ increased with the number of dives, with an average of 10.12% (95% CI, 8.29-11.95) of predicted values per 1,000 dives (*P* < .001). There was no significant difference in using 1 gas mixture over another or using different apparatuses.

##### FVC

FVC significantly increased with the number of dives, with an average increase of 3.02% of predicted values per 1,000 dives (95% CI, 2.52-3.53; *P* < .001). There was no significant difference in using 1 gas mixture over another or using different apparatuses. A higher BMI was associated with a larger FVC (0.51% per kg/m^2^, *P* = .010).

##### FEV_1_/FVC

FEV_1_/FVC was not influenced by the number of dives performed (*P* = .434). There was no significant difference in using 1 gas mixture over another, with widely overlapping CIs.

## Discussion

### Key Results

Mean FEV_1_ values at baseline perfectly fit predicted values (100.00%), indicating that the sample was comparable with the population used to create the equations for these predicted values. The multivariate analysis performed in this study showed a change of 3.21% in predicted values for 1,000 dives, after correcting for the effects of BMI, atopic history, and tobacco use on FEV_1_. This same association was found for other spirometric parameters used to assess ventilatory flows such as FEF_75%_ (10.12%) and for assessing lung volumes with FVC (3.02%). These variations were not greater than expected variability in the general population,^21^ but the whole cohort was impacted. Only the FEV_1_/FVC ratio was not influenced by diving because it was the ratio of 2 variables rising at the same pace. The increase in FEF_75%_ must be interpreted while keeping in mind that these values are measured at higher volumes when FVC is greater. The observed increase in FVC, FEV_1_, and FEF_75%_, expressed as % predicted values, should be interpreted with caution. In absolute values (without accounting for age, as presented in Table 2) and over the course of their career, FVC remained stable (95% CI, 5.32-5.48 L at baseline and 95% CI, 5.30-5.50 L at the end of follow-up), whereas flow rates decreased, with a reduction in FEV_1_ (95% CI, 4.54-4.66 L at baseline and 95% CI, 4.24-4.36 L at the end of follow-up) and FEF_75%_ (95% CI, 2.60-2.80 L at baseline and 95% CI, 1.80-2.00 L at the end of follow-up). The distinct types of gas and devices used do not significantly influence the evolution of FEV_1_ or other spirometric values. Thus, we cannot demonstrate a link between the oxygen concentration in the breathed mixture and lung function (dose effect). However, because the depth limits for diving are calculated based on the partial pressure of gases and not on their concentration in the breathed mixture, the dose effect of oxygen measured in partial pressure could be evaluated by studies with data collection allowing precise knowledge of the duration of each dive, their depth, and the mixture used. The discrepancy in our results compared with previous studies can be explained by adjusting the measured values for the age of the divers using predicted values. Indeed, we observe a decrease in ventilatory flows over time when using raw spirometric values measured in liters during consultations. Using predicted values ensures this decrease is smaller than expected due to ageing alone.

Tobacco consumption is known to affect lung function. We do not show any alteration of FEV_1_ associated with smoking in our study, but FEF_75%_ decreased with the extent of smoking history (−1.12% per pack-year), indicating that the cohort may be insufficient to demonstrate the impact of smoking on FEV_1_ (low power, which could have been improved by including more patients). A second hypothesis is that tobacco use impact may be reduced in this cohort compared with the general population due to physical training intensity.^22^ BMI is associated with a moderate FEV_1_ increase of 0.38% and a FVC increase of 0.51% predicted values per BMI point. This minimal, although significant, relationship between BMI and volume is to be expected given the minimal changes in BMI over time.^23^ Interestingly, the direction of change is typically lower not higher, and it may be that the BMI increase in this particular population is a reflection of greater muscle mass as opposed to sedentary behavior. Atopic status does not significantly modify spirometric values measured in this cohort. The exclusion of symptomatic patients with asthma from the cohort due to their inability to engage could explain the weak and nonsignificant impact of atopy on lung function in this cohort. Among the patients included, despite their atopic status or even asthma, the hidden use of ß2-mimetics in anticipation of fitness visits could also explain the lack of significance. Finally, a lack of power (not enough patients) may also be responsible for the lack of significant effect of atopy on FEV_1_ and FEF_75%_.

### Limitations

Despite its strengths, this study has some important limitations to consider. First, the use of predicted value cannot completely replace an adequate control group. The studied sample consists exclusively of military personnel, meaning the results may not be generalizable to a broader population of professional divers because military diving practices are specific and distinct from those of civilian professional divers. Military diving equipment, protocols, and physical training differ significantly from civilian divers, which could uniquely influence the outcomes. There is currently no database to track civilian professional divers; therefore, the comparability of results is limited. Additionally, the absence of female participants in the sample limits the scope of the conclusions. Furthermore, we cannot establish a dose-response relationship between oxygen exposure and its effects on FEV_1_ because the various gas mixtures used similarly impact this measure. Dive characteristics (eg, duration, depth, temperature) could not be evaluated due to their strong interdependence with the types of gases and equipment used and due to the quality of collected data. For instance, closed-circuit oxygen divers undertake longer but shallower dives than others. Including duration and depth in the analysis model in addition to the gas mixture could have led us to mistakenly conclude either the absence of an effect of these 3 parameters or attribute the effects of 1 parameter to the others. These results do not allow for the affirmation of a causal relationship between diving and the increase in FEV_1_. There is merely a correlation between these 2 variables, indicating that the FEV_1_ of divers tends to increase compared with the general population as they undertake more dives. It would be premature to attribute this increase directly to diving. More in-depth analyses of this database are necessary to understand better the long-term effects of diving on pulmonary function. A better interpretation of spirometric values evolution could have been made with *z* scores instead of % predicted values. Our initial analysis plan did not consider this opportunity of using *z* scores but preferred comparability of results with previous studies using % predicted values.

Although these limitations must be considered, the methodology of this study demonstrates considerable improvements over earlier retrospective studies, particularly through the inclusion of more spirometric measurements and longer follow-up.^7^^,^^24^ Predicted values account for age, height, sex, and ethnicity, whereas the model adjusts for variables known to impact lung function (BMI, atopic history, tobacco use history, socioprofessional category) and accounts for different dive characteristics.^25^^,^^26^ Thus, to our knowledge, this is the first study to evaluate differences between air, NITROX, and pure oxygen.^7^ However, military divers are offered diverse career paths, leading them to specialties requiring various intensities of physical training and levels of adverse occupational and environmental exposures (eg, moisture or asbestos in navy ships, leptospirosis or other infectious diseases met during missions and training).^5^ Water and inhaled gas temperature and depth and length of dives could not be evaluated due to the collinearity with the type of gas and apparatus used.

Although this study aimed to explore the impact of diving on lung function in French military divers, these other factors may also influence lung function evolution. The predicted values offer the possibility of accounting for normal ageing but do not account for these factors, and the retrospective design of this study does not allow for adequate collection of these factors.^27^ These factors may influence FEV_1_ on a more important scale than SCUBA diving. The sample of divers included in the study may not sufficiently represent the overall population used to create the equations for predicted values. Still, this hypothesis is not supported by the average value of FEV_1_ at baseline (100.00% of predicted value) when the patients were already enrolled in the military and physically fit and trained. Another hypothesis is that diving may strengthen respiratory muscles due to the higher airflow resistances met when breathing with SCUBA equipment and the higher respiratory effort needed. This is supported by the positive effect that respiratory muscle training has on lung function.^28^, ^29^, ^30^ However, the studies focused on the impact on lung function of patients with neurologic diseases, asthma, or COPD. Thus, the impact of respiratory muscle training on healthy patients is unclear. Finally, the spirometric test instructions may be better understood by patients at the end of the follow-up than at the first lung function assessment, artificially increasing the values over time.^31^

## Interpretation

SCUBA diving was associated with a slower decline of pulmonary flows and volumes over time in this population of military divers. However, this association should not be interpreted as evidence that diving is directly responsible for these changes because it contrasts with findings from previous studies and many potential confounders were not explored. Our findings emphasize the importance of further investigation into the long-term effects of underwater diving on pulmonary function among military personnel. Prospective studies incorporating nondiving control patients could more precisely explore the unmeasured confounding factors in our study. These studies could significantly contribute to health policies for both military and civilian divers by enhancing our understanding of the impact of diving on their respiratory health. As a result, the modalities of pulmonary function monitoring could be adapted, including improving the quality of spirometry tests through better explanations and instructions for performance, and extending follow-up beyond the professional career.

## Funding/Support

The authors have reported to *CHEST Pulmonary* that no funding was received for this study.

## Financial/Nonfinancial Disclosures

None declared.
